# Higher order Riesz transforms for Hermite expansions

**DOI:** 10.1186/s13660-017-1376-1

**Published:** 2017-05-03

**Authors:** Jizheng Huang

**Affiliations:** College of Sciences, North China University of Technology, Beijing, 100144 China

**Keywords:** 42C10, 42B25, Hermite expansions, Littlewood-Paley *g*-function, Riesz transform, Hardy space

## Abstract

In this paper, we consider the Riesz transform of higher order associated with the harmonic oscillator $L=-\Delta+|x|^{2}$, where Δ is the Laplacian on $\mathbb {R}^{d}$. Moreover, the boundedness of Riesz transforms of higher order associated with Hermite functions on the Hardy space is proved.

## Introduction

Let $H_{k}(x)$ denote the Hermite polynomials on $\mathbb {R}$, which can be defined as

$$H_{k}(x)=(-1)^{k}\frac{d^{k}}{dx^{k}}\bigl(e^{-x^{2}} \bigr)e^{x^{2}},\quad k=0,1,2,\ldots. $$

The normalized Hermite functions are defined by

$$h_{k}(x)= \bigl(\pi^{1/2}2^{k}k! \bigr)^{-1/2}H_{k}(x)\exp \bigl(-x^{2}/2 \bigr),\quad k=0,1,\ldots. $$

The high dimensional Hermite functions on $\mathbb{R}^{d}$ can be defined in the following way. For $\alpha=(\alpha_{1},\ldots, \alpha_{d})$, $\alpha_{i}\in \{0,1,\ldots\}$, $x=(x_{1},\ldots, x_{d})\in\mathbb{R}^{d}$,

$$h_{\alpha}(x)=\prod_{j=1}^{d} h_{\alpha_{j}}(x_{j}). $$

$\{h_{\alpha}\}$ forms a complete orthonormal basis of $L^{2}(\mathbb{R}^{d})$. Let $|\alpha |=\alpha_{1}+\cdots+\alpha_{d}$, then we have

$$Lh_{\alpha}= \bigl(2 \vert \alpha \vert +d \bigr)h_{\alpha}. $$

A very famous reference for Hermite functions is [1].

The operator *L* is positive and symmetric on $L^{2}(\mathbb {R}^{d})$. Let $\{T_{t}^{L}\}_{t\geq0}$ be the heat kernel defined by

$$T_{t}^{L}f=e^{-tL}f=\sum _{n=0}^{\infty}e^{-t(2n+d)}\mathcal{P}_{n} f $$

for $f\in L^{2}(\mathbb{R}^{d})$ and

$$\mathcal{P}_{n}f=\sum_{|\alpha|=n}\langle f, h_{\alpha}\rangle h_{\alpha}. $$

Then the Poisson semigroup is defined as

$$P_{t}^{L}f=e^{-tL^{1/2}}f=\sum _{n=0}^{\infty}e^{-t(2n+d)^{1/2}}\mathcal{P}_{n} f, \quad f\in L^{2}\bigl(\mathbb{R}^{d}\bigr). $$

The relation between the heat kernel and the Poisson kernel is

1 $$ P_{t}^{L}f(x)=\frac{t}{\sqrt{4\pi}} \int_{0}^{\infty}s^{-3/2}\exp \bigl(-t^{2}/4s \bigr)T_{s}^{L}f(x)\,ds. $$

Let $A_{j}=\frac{\partial}{\partial x_{j}}+x_{j}$ and $A_{-j}=A^{*}_{j}=-\frac {\partial}{\partial x_{j}}+x_{j}$, $j=1,2,\ldots,d$. Then we can denote *L* as

$$L=-\frac{1}{2} \bigl[ (\nabla+x )\cdot (\nabla-x )+ (\nabla -x )\cdot ( \nabla+x ) \bigr]=\frac{1}{2}\sum_{j=1}^{d}A_{j}A_{-j}+A_{-j}A_{j}. $$

We define operators $R_{\pm j}^{L}$, $j=1,2,\ldots,d$

$$R_{j}^{L}=A_{j}L^{-1/2},\qquad R_{-j}^{L}=A_{-j}L^{-1/2}. $$

$R_{j}$ and $R_{-j}$ are called the Riesz transforms associated with *L*. The definition was first suggested by Thangavelu in [2].

Let $e_{j}$ be the coordinate vectors in $\mathbb{R}^{d}$, then

$$A_{j}h_{\alpha}=(2\alpha_{j}+2)^{1/2}h_{\alpha+e_{j}}, \qquad A_{-j}h_{\alpha}=(2\alpha _{j})^{1/2}h_{\alpha-e_{j}}. $$

Therefore, for $f\in L^{2}(\mathbb{R}^{d})$,

2 $$\begin{aligned} R_{j}^{L}f =&\sum_{\alpha}\biggl(\frac{2\alpha_{j}}{2|\alpha|+d} \biggr)^{1/2} \langle f, h_{\alpha}\rangle h_{\alpha-e_{j}} \\ =&\sum_{n=0}^{\infty}\sum _{|\alpha|=n} \biggl(\frac{2\alpha _{j}}{2n+d} \biggr)^{1/2} \langle f, h_{\alpha}\rangle h_{\alpha-e_{j}}, \end{aligned}$$

and

3 $$\begin{aligned} R_{-j}^{L}f =&\sum_{\alpha}\biggl(\frac{2(\alpha_{j}+1)}{2|\alpha|+d} \biggr)^{1/2} \langle f, h_{\alpha}\rangle h_{\alpha+e_{j}} \\ =&\sum_{n=0}^{\infty}\sum _{|\alpha|=n} \biggl(\frac{2(\alpha _{j}+1)}{2n+d} \biggr)^{1/2} \langle f, h_{\alpha}\rangle h_{\alpha+e_{j}}. \end{aligned}$$

In [1], the author proved that $R_{j}^{L}$ were bounded on the local Hardy spaces $h^{1}(\mathbb {R}^{d})$ which were defined by Goldberg in [3]. Thangavelu asked one question: whether it was possible to characterize $h^{1}(\mathbb {R}^{d})$ by $R_{j}^{L}$, i.e., whether the equality

$$h^{1}\bigl(\mathbb {R}^{d}\bigr)=\bigl\{ f\in L^{1} \bigl(\mathbb {R}^{d}\bigr): R_{j}^{L}f\in L^{1}\bigl(\mathbb {R}^{d}\bigr), j=1,2,\ldots,d\bigr\} $$

is true. In [4], the author proved the boundedness of $R_{j}^{L}$ on Hardy spaces $H_{L}^{1}(\mathbb{R}^{d})$, $d\geq3$, where $H_{L}^{1}(\mathbb{R}^{d})$ are the Hardy spaces for *L* (cf. [5]).

### Proposition 1

*Let* $j=1,2,\ldots,d$. *Then the operators* $R_{j}^{L}$ *are bounded on* $H_{L}^{1}(\mathbb {R}^{d})$, *that is*, *there exists* $C>0$ *satisfying*

$$\bigl\Vert R_{j}^{L}f \bigr\Vert _{H_{L}^{1}}\leq C \Vert f \Vert _{H_{L}^{1}}. $$

Moreover, he characterized $H_{L}^{1}(\mathbb{R}^{d})$ by $R_{j}^{L}$, $j=1,2,\ldots,d$. Therefore, we cannot characterize $h^{1}(\mathbb {R}^{d})$ by $R_{j}^{L}$.

### Remark 1

When we consider the boundedness of Riesz transforms for *L* on Hardy spaces, the main tool is Littlewood-Paley characterizations of Hardy spaces. In fact, we have the following equality (cf. [4]):

$$t\partial_{t}e^{-t(L\pm2)^{1/2}} \bigl(R_{\pm j}^{L}f \bigr)=-t \biggl(\pm\frac {\partial}{\partial x_{j}}+x_{j} \biggr)e^{-tL^{1/2}}f $$

for all $j=1,2,\ldots,d$ and $f\in L^{2}(\mathbb{R}^{d})$. If we prove the boundedness of Riesz transforms $R_{-j}^{L}$ on Hardy spaces, we need to consider the operator $L-2$. Since the Hardy spaces $H_{L}^{1}(\mathbb{R}^{d})$, $d\geq3$, associated with *L* defined in [5] are for nonnegative potentials, it is maybe natural to just consider $R_{j}^{L}$. In [6], the authors proved the boundedness of $R_{\pm j}^{L}$ on $L^{p}(\mathbb {R}^{d})$, where they considered the semigroup generated by $L+b$ for $b<0$ on $L^{p}(\mathbb {R}^{d})$.

In this paper, we prove that the higher ordered Riesz transforms are bounded on the Hardy spaces associated with Hermite functions. More precisely, let

$$L^{-m/2}h_{\alpha}=\bigl(2 \vert \alpha \vert +d \bigr)^{-m/2}h_{\alpha}, $$

and define the *m*-ordered Riesz transforms as

$$R_{i_{1}i_{2}\cdots i_{m}}=A_{i_{1}}A_{i_{2}}\cdots A_{i_{m}}L^{-m/2}, $$

where $1\leq i_{j}\leq d$ and $1\leq j\leq m$.

We define Hardy space $H_{L}^{1}(\mathbb{R}^{d})$ for $d\geq3$ as follows (cf. [5]):

$$H_{L}^{1}\bigl(\mathbb{R}^{d}\bigr)= \bigl\{ f\in L^{1}\bigl(\mathbb{R}^{d}\bigr): \mathcal{M}_{L}f \in L^{1}\bigl(\mathbb{R}^{d}\bigr) \bigr\} , $$

where $\mathcal{M}_{L}f(x)=\sup_{t>0}|T_{t}^{L}f(x)|$.

Define

4 $$ \rho(x)=\frac{1}{1+|x|}, $$

we say $a(x)$ is an atom for the space $H_{L}^{1}(\mathbb {R}^{d})$ if there exists a ball $B(x_{0},r)$ such that

1. $\operatorname{supp} a\subset B(x_{0},r)$,
2. $\|a\|_{L^{\infty}}\leq|B(x_{0},r)|^{-1}$,
3. if $r<\rho(x_{0})$, then $\int a(x) \,dx=0$.

The atomic quasi-norm in $H_{L}^{1}(\mathbb {R}^{d})$ can be defined as

$$\|f\|_{L\text{-atom}}=\inf \Bigl\{ \sum|c_{j}| \Bigr\} . $$

In [5], the authors proved the following result.

### Proposition 2

*There exists* $C>0$ *satisfying*

$$C^{-1}\|f\|_{H_{L}^{1}}\leq\|f\|_{L\textit{-atom}}\leq C \|f \|_{H_{L}^{1}}. $$

Let $b\in\mathbb {R}_{+}^{d}$. We define

$$G_{t}^{b}(x,y)=e^{-bt}G_{t}^{L}(x,y). $$

Then

$$G_{t}^{b}(f) (x)= \int_{\mathbb {R}^{d}}G_{t}^{b}(x,y)f(y)\,dy $$

is a semigroup for the spaces $L^{p}(\mathbb {R}^{d})$, $1\leq p<\infty$, and $\|G_{t}^{b}(f)\|_{L^{p}(\mathbb {R}^{d})}\leq e^{-bt}\|f\|_{L^{p}(\mathbb {R}^{d})}$. This semigroup is generated by the operator $-(L+b)$.

The subordination formula is

5 $$ P_{t}^{b}(x,y)=\frac{t}{\sqrt{4\pi}} \int_{0}^{\infty }G_{s}^{b}(x,y)s^{-3/2}e^{-t^{2}/4s} \,ds. $$

The Poisson integral of $f(x)$ can be defined as

$$\begin{aligned} u_{b}(x,t) =&P_{t}^{b}(f) (x)= \int_{\mathbb {R}^{d}}P_{t}^{b}(x,y)f(y)\,dy \\ =&\frac{t}{\sqrt{4\pi}} \int_{\mathbb {R}^{d}} \int_{0}^{\infty }G_{s}^{b}(x,y)f(y)s^{-3/2}e^{-t^{2}/4s} \,ds\,dy. \end{aligned}$$

Let

$$\mathcal{G}_{b}(f) (x)= \Biggl( \int_{0}^{\infty}\sum_{j=0}^{d} \bigl\vert tA_{j}u_{b}(x,t) \bigr\vert ^{2} \frac{dt}{t} \Biggr)^{1/2} $$

and

$$\mathcal{G}_{b}^{1}(f) (x)= \biggl( \int_{0}^{\infty}\bigl\vert t\partial_{t}u_{b}(x,t) \bigr\vert ^{2}\frac{dt}{t} \biggr)^{1/2}, $$

where $A_{0}=\partial_{t}$.

The main results of this paper are as follows.

### Theorem 1

$f\in H_{L}^{1}(\mathbb {R}^{d})$ *is equivalent to* $\mathcal{G}_{b}(f)\in L^{1}(\mathbb {R}^{d})$ *and* $f\in L^{1}(\mathbb{R}^{d})$. *Moreover*,

$$\Vert f \Vert _{H_{L}^{1}}\sim \bigl\Vert \mathcal{G}_{b}(f) \bigr\Vert _{L^{1}}+ \Vert f \Vert _{L^{1}}. $$

### Theorem 2

*The operators* $R_{i_{1}i_{2}\cdots i_{m}}=A_{i_{1}}A_{i_{2}}\cdots A_{i_{m}}L^{-m/2}$ *are bounded on* $H_{L}^{1}(\mathbb {R}^{d})$ *for all* $1\leq i_{j}\leq d$ *for every* $1\leq j\leq m$, *that is*, *there exists* $C>0$ *satisfying*

$$\Vert R_{i_{1}i_{2}\cdots i_{m}}f \Vert _{H_{L}^{1}}\leq C \Vert f \Vert _{H_{L}^{1}}. $$

The organization of this paper is as follows. In Section 2, we give some estimations of the heat kernel and the Poisson kernel associated with $L+b$. In Section 3, Theorem 1 is proved. In Section 4, we prove Theorem 2.

Throughout the article, we use *A* and *C* to denote the positive constants, which are independent of the main parameters and may be different at each occurrence. By $B_{1} \sim B_{2}$, we mean that there exists a constant $C>1$ such that $\frac{1}{C} \leq \frac{B_{1}}{B_{2}}\leq C$.

## Estimations of the kernels

Let $G_{t}^{b}(x,y)$ be the heat kernel of $\{T_{t}^{L+b}\}$. Then the following inequality can be proved by the Feynman-Kac formula:

$$G_{t}^{b}(x,y)\leq W_{t}(x-y), $$

where

$$W_{t}(x)= (4\pi t )^{-d/2}\exp \bigl(-|x|^{2}/(4t) \bigr) $$

is the heat kernel on $\mathbb{R}^{d}$.

Since $G_{t}^{b}(x,y)\leq G_{t}^{L}(x,y)$, we have (cf. [7]) the following lemma.

### Lemma 1

- *For* $N \in\mathbb {N}$, *there exists* $C_{N}>0$
  6 $$ 0\leq G_{t}^{b}(x,y)\leq C_{N}t^{-\frac{d}{2}}e^{-(5t)^{-1}|x-y|^{2}} \biggl(1+\frac{\sqrt{t}}{\rho(x)}+\frac{\sqrt{t}}{\rho(y)} \biggr)^{-N}. $$
- *There are constants* $0<\delta<1$ *and* $C>0$, *for* $N>0$, *there is* $C_{N}>0$ *which satisfies for all* $|h|\leq \frac{|x-y|}{2}$,
  7 $$ \bigl\vert G_{t}^{b}(x+h,y)-G_{t}^{b}(x,y) \bigr\vert \leq C_{N} \biggl(\frac{|h|}{\sqrt{t}} \biggr)^{\delta}t^{-\frac{d}{2}} e^{-At^{-1}|x-y|^{2}} \biggl(1+ \frac{\sqrt{t}}{\rho(x)}+\frac{\sqrt {t}}{\rho(y)} \biggr)^{-N}. $$

By the subordination formula, we get the following.

### Lemma 2

- *For* $N\in\mathbb {N}$, *there is* $C_{N}>0$ *satisfying*
  8 $$ 0\leq P_{t}^{b}(x,y)\leq C_{N} \frac{t}{(t^{2}+A|x-y|^{2})^{(d+1)/2}} \biggl(1+\frac{t}{\rho(x)}+\frac{t}{\rho(y)} \biggr)^{-N}. $$
- *Let* $0<\delta<1$ *and* $|h|<\frac{|x-y|}{2}$. *Then*, *for* $N\in\mathbb {N}$, *there are* $C>0$, $C_{N}>0$ *satisfying*
  9 $$\begin{aligned}& \bigl\vert P_{t}^{b}(x+h,y)-P_{t}^{b}(x,y) \bigr\vert \\& \quad \leq C_{N} \biggl(\frac{|h|}{t} \biggr)^{\delta}\frac{t}{(t^{2}+A|x-y|^{2})^{(d+1)/2}} \biggl(1+\frac{t}{\rho(x)}+ \frac{t}{\rho(y)} \biggr)^{-N}. \end{aligned}$$

### Proof

(a) By subordination formula and Lemma 1, we have

10 $$\begin{aligned} 0 \leq& P_{t}^{b}(x,y)=\frac{1}{\sqrt{\pi}} \int_{0}^{\infty}G_{t^{2}/4\mu }^{b}(x,y)e^{-\mu} \mu^{-1/2}\,d\mu \\ \leq&C_{N} \int_{0}^{\infty} \biggl(\frac{t^{2}}{4\mu} \biggr)^{-\frac {d}{2}}e^{-C_{1} t^{-2}(4\mu|x-y|^{2})} \biggl(\frac{t}{\sqrt{4\mu}\rho(x)}+ \frac{t}{\sqrt{4\mu}\rho(y)} \biggr)^{-N}e^{-\mu}\mu^{-1/2}\,d\mu \\ =&C_{N} \int_{0}^{\infty} \biggl(\frac{t^{2}}{4\mu} \biggr)^{-\frac {d}{2}}e^{-C_{1} t^{-2}(4\mu|x-y|^{2})} \biggl(\frac{t}{\rho(x)}+ \frac{t}{\rho(y)} \biggr)^{-N}e^{-\mu}\mu ^{N/2-1/2}\,d\mu \\ \leq&C_{N} \biggl(\frac{t}{\rho(x)}+\frac{t}{\rho(y)} \biggr)^{-N} \int_{0}^{\infty} \biggl(\frac{t^{2}}{4\mu} \biggr)^{-\frac{d}{2}}e^{-C_{1} t^{-2}(4\mu|x-y|^{2})} e^{-\mu}\mu^{-1/2}\,d\mu \\ =&C_{N} \biggl(\frac{t}{\rho(x)}+\frac{t}{\rho(y)} \biggr)^{-N}\frac {t}{(t^{2}+A|x-y|^{2})^{(d+1)/2}} . \end{aligned}$$

By (10) and

$$P_{t}^{b}(x,y)\leq\frac{t}{(t^{2}+A|x-y|^{2})^{(d+1)/2}}, $$

we get

$$0\leq P_{t}^{b}(x,y)\leq C_{N} \frac{t}{(t^{2}+A|x-y|^{2})^{(d+1)/2}} \biggl(1+\frac{t}{\rho(x)}+\frac{t}{\rho(y)} \biggr)^{-N}. $$

(b) By subordination formula again, we know

11 $$\begin{aligned}& \bigl\vert P_{t}^{b}(x+h,y)-P_{t}^{b}(x,y) \bigr\vert \\& \quad \leq \frac{1}{\sqrt{\pi}} \int_{0}^{\infty} \bigl\vert G_{t^{2}/4\mu }^{b}(x+h,y)-G_{t^{2}/4\mu}^{b}(x,y) \bigr\vert e^{-\mu}\mu^{-1/2}\,d\mu \\& \quad \leq C_{N} \int_{0}^{\infty}\biggl(\frac{t^{2}}{4\mu} \biggr)^{-\frac {d}{2}}e^{-C_{1} t^{-2}(4\mu|x-y|^{2})} \biggl(\frac{t}{\sqrt{4\mu}\rho(x)}+ \frac{t}{\sqrt{4\mu}\rho(y)} \biggr)^{-N} \\& \qquad {}\times\biggl(\frac{\sqrt{4\mu}|h|}{t}\biggr)^{\delta'}e^{-\mu} \mu^{-1/2}\,d\mu \\& \quad = C_{N}\biggl(\frac{|h|}{t}\biggr)^{\delta'} \biggl( \frac{t}{\rho(x)}+\frac {t}{\rho(y)} \biggr)^{-N} \int_{0}^{\infty}\biggl(\frac{t^{2}}{4\mu} \biggr)^{-\frac{d}{2}} e^{-C_{1} t^{-2}(4\mu|x-y|^{2})}e^{-\mu}\mu^{(N+\delta')/2-1/2}\,d\mu \\& \quad \leq C_{N} \biggl(\frac{|h|}{t}\biggr)^{\delta'} \biggl(\frac{t}{\rho (x)}+\frac{t}{\rho(y)} \biggr)^{-N} \int_{0}^{\infty}\biggl(\frac{t^{2}}{4\mu} \biggr)^{-\frac{d}{2}}e^{-C'_{1} t^{-2}(4\mu|x-y|^{2})} e^{-\mu}\mu^{-1/2}\,d\mu \\& \quad = C_{N} \biggl(\frac{|h|}{t}\biggr)^{\delta'} \biggl( \frac{t}{\rho(x)}+\frac {t}{\rho(y)} \biggr)^{-N} \frac{t}{(t^{2}+A|x-y|^{2})^{(d+1)/2}} . \end{aligned}$$

We also have

12 $$\begin{aligned}& \bigl\vert P_{t}^{b}(x+h,y)-P_{t}^{b}(x,y) \bigr\vert \\& \quad \leq C_{N} \int_{0}^{\infty}\biggl(\frac{t^{2}}{4\mu} \biggr)^{-\frac {d}{2}}e^{-C_{1}t^{-2}(4\mu|x-y|^{2})} \biggl(\frac{\sqrt{4\mu}|h|}{t} \biggr)^{\delta'}e^{-\mu}\mu^{-1/2}\,d\mu \\& \quad = C_{N}\biggl(\frac{|h|}{t}\biggr)^{\delta'} \int_{0}^{\infty}\biggl(\frac{t^{2}}{4\mu} \biggr)^{-\frac{d}{2}} e^{-C_{1} t^{-2}(4\mu|x-y|^{2})}e^{-\mu}\mu^{\delta'/2-1/2}\,d\mu \\& \quad \leq C_{N} \biggl(\frac{|h|}{t}\biggr)^{\delta'} \int_{0}^{\infty}\biggl(\frac{t^{2}}{4\mu} \biggr)^{-\frac{d}{2}}e^{-C'_{1} t^{-2}(4\mu|x-y|^{2})} e^{-\mu}\mu^{-1/2}\,d\mu \\& \quad = C_{N} \biggl(\frac{|h|}{t}\biggr)^{\delta'} \frac{t}{(t^{2}+A|x-y|^{2})^{(d+1)/2}} . \end{aligned}$$

Then (b) follows from (11) and (12). □

Let $D_{t}^{b,k}(x,y)=t^{k}\partial_{t}^{k}P_{t}^{b}(x,y)$. Then, by Lemma 2, we can prove (cf. [8] or [9]) the following.

### Proposition 3

*There are* $C>0$, $0<\delta'<\delta$, *for* $N\in\mathbb {N}$, *there is* $C_{N}$ *such that*

$$\begin{aligned} (\mathrm{a}) &\quad \bigl\vert D_{t}^{b,k}(x,y) \bigr\vert \leq C_{N}\frac {t}{(t^{2}+C|x-y|^{2})^{(d+1)/2}} \biggl(1+\frac{t}{\rho(x)}+ \frac{t}{\rho (y)} \biggr)^{-N} ; \\ (\mathrm{b}) &\quad \bigl\vert D_{t}^{b,k}(x+h,y)-D_{t}^{b,k}(x,y) \bigr\vert \\ &\quad \quad \leq C_{N} \biggl(\frac{|h|}{t} \biggr)^{\delta'}\frac {t}{(t^{2}+C|x-y|^{2})^{(d+1)/2}} \biggl(1+\frac{t}{\rho(x)}+ \frac{t}{\rho (y)} \biggr)^{-N} \\ &\quad \qquad \textit{for all } |h|\leq\frac{|x-y|}{2}. \end{aligned}$$

Let $t=\frac{1}{2}\ln\frac{1+s}{1-s}$, $s\in(0,1)$. Then

13 $$ G_{t}(x,y)= \biggl(\frac{1-s^{2}}{4\pi s} \biggr)^{d/2}\exp{ \biggl(-\frac {1}{4}\biggl(s|x+y|^{2}+ \frac{1}{s}|x-y|^{2}\biggr) \biggr)}\doteq K_{s}(x,y). $$

The proof of the following proposition is motivated by [10].

### Proposition 4

*There is* $A>0$, *for* $N\in\mathbb {N}$ *and* $|x-x'|\leq\frac{|x-y|}{2}$, *we can find* $C_{N}>0$ *such that*

$$\begin{aligned} (\mathrm{a})&\quad \bigl\vert tA_{j}G_{t}^{b}(x,y) \bigr\vert \leq C_{N}t^{-\frac{d}{2}} \exp{ \biggl(- \frac{|x-y|^{2}}{At} \biggr)} \biggl(1+\frac{\sqrt{t}}{\rho(x)}+\frac{\sqrt {t}}{\rho(y)} \biggr)^{-N} ; \\ (\mathrm{b})&\quad \bigl\vert tA_{j}G_{t}^{b}(x,y)-tA_{j}G_{t}^{b} \bigl(x',y\bigr) \bigr\vert \\ &\quad \quad \leq C_{N} \frac{|x-x'|}{t}t^{-\frac{d}{2}}\exp{ \biggl(-\frac {|x-y|^{2}}{At} \biggr)} \biggl(1+\frac{\sqrt{t}}{\rho(x)}+\frac{\sqrt {t}}{\rho(y)} \biggr)^{-N}. \end{aligned}$$

### Proof

By

$$\begin{aligned} \begin{aligned} \bigl\vert A_{j}G_{t}(x,y) \bigr\vert &= \biggl\vert \frac{\partial}{\partial x_{j}}G_{t}(x,y)+x_{j}G_{t}(x,y) \biggr\vert \\ &\leq \biggl\vert \frac{\partial}{\partial x_{j}}G_{t}(x,y) \biggr\vert + \bigl\vert x_{j}G_{t}(x,y) \bigr\vert \doteq I_{1}+I_{2}, \end{aligned} \end{aligned}$$

and $t=\frac{1}{2}\ln\frac{1+s}{1-s}\sim s$, $s\rightarrow0^{+}$, for $s\in (0,\frac{1}{2}]$, we have

$$\begin{aligned} I_{2} \le&C|x_{j}|s^{-\frac{d}{2}}\exp{ \biggl(- \frac{1}{4}s|x+y|^{2} \biggr)}\exp{ \biggl(-\frac{1}{4} \frac{|x-y|^{2}}{s} \biggr)} \\ \leq&C|x|s^{-\frac{d}{2}}\exp{ \biggl(-\frac{1}{4}s|x+y|^{2} \biggr)}\exp { \biggl(-\frac{1}{4}\frac{|x-y|^{2}}{s} \biggr)}. \end{aligned}$$

If $x\cdot y\leq0$, then $|x|\leq|x-y|$. So

$$\begin{aligned} I_{2} \le&Cs^{-\frac{d}{2}}|x-y|\exp{ \biggl(-\frac{1}{4} \frac {|x-y|^{2}}{s} \biggr)}\leq C s^{-\frac{d-1}{2}}\exp{ \biggl(-\frac {|x-y|^{2}}{8s} \biggr)} \\ \leq&C t^{-\frac{d-1}{2}}\exp{ \biggl(-\frac{|x-y|^{2}}{8t} \biggr)}. \end{aligned}$$

If $x\cdot y\geq0$, then $|x|\leq|x+y|$. So

$$\begin{aligned} I_{2} \le&Cs^{-\frac{d}{2}}|x+y|\exp{ \biggl(-\frac{1}{4}s|x+y|^{2} \biggr)}\exp{ \biggl(-\frac{1}{4}\frac{|x-y|^{2}}{s} \biggr)} \\ \leq& C s^{-\frac{d+1}{2}}\exp{ \biggl(-\frac{|x-y|^{2}}{4s} \biggr)} \leq C t^{-\frac{d+1}{2}}\exp{ \biggl(-\frac{|x-y|^{2}}{4t} \biggr)}. \end{aligned}$$

Therefore,

14 $$ |tI_{2}|\leq C\bigl(t^{\frac{3}{2}}+t^{\frac{1}{2}} \bigr)e^{-bt}t^{-\frac{d}{2}}\exp { \biggl(-\frac{|x-y|^{2}}{8t} \biggr)} \leq Ct^{-\frac{d}{2}}\exp{ \biggl(-\frac{|x-y|^{2}}{8t} \biggr)}. $$

When $s\in[\frac{1}{2},1)$,

$$\begin{aligned} I_{2} \leq&C|x_{j}|\exp{ \biggl(-\frac{1}{4} \biggl(s|x+y|^{2}+\frac{|x-y|^{2}}{s}\biggr) \biggr)} \\ \leq&C|x|s^{-\frac{d}{2}}\exp{ \biggl(-\frac{1}{4}\biggl(s|x+y|^{2}+ \frac {|x-y|^{2}}{s}\biggr) \biggr)} \\ \leq&C(|x+y|+|x-y|)s^{-\frac{d}{2}}\exp{ \biggl(-\frac {1}{4} \biggl(s|x+y|^{2}+\frac{|x-y|^{2}}{s}\biggr) \biggr)} \\ \leq&C\exp{ \biggl(-\frac{|x-y|^{2}}{8s} \biggr)}. \end{aligned}$$

Since $t=\frac{1}{2}\ln\frac{1+s}{1-s}> s$ for $s\in[\frac{1}{2},1)$, we get

$$I_{2}\leq C\exp{ \biggl(-\frac{|x-y|^{2}}{8t} \biggr)}. $$

Therefore,

15 $$ |tI_{2}|\leq Cte^{-bt}\exp{ \biggl(- \frac{|x-y|^{2}}{8t} \biggr)} \leq Ct^{-\frac{d}{2}}\exp{ \biggl(-\frac{|x-y|^{2}}{8t} \biggr)}. $$

By (13), we get

$$\frac{\partial}{\partial x_{j}}K_{s}(x,y)=-\frac{1}{2}\biggl(s(x_{j}+y_{j})+ \frac {1}{s}(x_{j}-y_{j})\biggr)K_{s}(x,y), $$

and

$$ I_{1}\leq C\biggl(s|x_{j}+y_{j}|+ \frac{1}{s}|x_{j}-y_{j}|\biggr)K_{s}(x,y) \leq C\biggl(s|x+y|+\frac {1}{s}|x-y|\biggr)K_{s}(x,y). $$

Therefore, when $s\in(0,\frac{1}{2}]$, we have

$$I_{1}\leq C s^{-\frac{d}{2}}\exp{ \biggl(-\frac{|x-y|^{2}}{8s} \biggr)} \leq Ct^{-\frac{d}{2}}\exp{ \biggl(-\frac{|x-y|^{2}}{8t} \biggr)}. $$

When $s\in[\frac{1}{2},1)$, we have

$$I_{1}\leq C \exp{ \biggl(-\frac{|x-y|^{2}}{8s} \biggr)}\leq C\exp{ \biggl(-\frac {|x-y|^{2}}{8t} \biggr)}. $$

Then

16 $$ \biggl\vert t\frac{\partial}{\partial x_{j}}G_{t}^{b}(x,y) \biggr\vert \leq Ct\bigl(1+t^{-\frac {d}{2}}\bigr)e^{-bt}\exp{ \biggl(- \frac{|x-y|^{2}}{8t} \biggr)} \leq Ct^{-\frac{d}{2}}\exp{ \biggl(-\frac{|x-y|^{2}}{8t} \biggr)}. $$

By (14)-(16), we get

17 $$ \bigl\vert tA_{j}G_{t}^{b}(x,y) \bigr\vert \leq Ct^{-\frac{d}{2}}\exp{ \biggl(-\frac{|x-y|^{2}}{8t} \biggr)}. $$

Similar to the proof of (17), for any $N>0$, we can prove

$$\bigl(\sqrt{t} \vert x \vert \bigr)^{N} \bigl\vert tA_{j}G_{t}^{b}(x,y) \bigr\vert \leq C_{N}t^{-\frac{d}{2}}\exp{ \biggl(-\frac {|x-y|^{2}}{8t} \biggr)} $$

and

$$t^{N} \bigl\vert tA_{j}G_{t}^{b}(x,y) \bigr\vert \leq C_{N}t^{-\frac{d}{2}}\exp{ \biggl(-\frac {|x-y|^{2}}{8t} \biggr)}. $$

Since $\rho(x)=\frac{1}{1+|x|}$, we get $\frac{\sqrt{t}}{\rho(x)}=\sqrt{t}(1+|x|)$. Then, for $N>0$,

18 $$ \biggl(\frac{\sqrt{t}}{\rho(x)} \biggr)^{N} \bigl\vert tA_{j}G_{t}^{b}(x,y) \bigr\vert \leq C_{N}t^{-\frac{d}{2}}\exp{ \biggl(-\frac{|x-y|^{2}}{8t} \biggr)}. $$

Since *x* and *y* are symmetric, we also have

19 $$ \biggl(\frac{\sqrt{t}}{\rho(y)} \biggr)^{N} \bigl\vert tA_{j}G_{t}^{b}(x,y) \bigr\vert \leq C_{N}t^{-\frac{d}{2}}\exp{ \biggl(-\frac{|x-y|^{2}}{8t} \biggr)}. $$

Then (a) follows from (17)-(19).

(b) Note that

$$\begin{aligned}& \bigl\vert tA_{j}G_{t}^{b} \bigl(x',y\bigr)-tA_{j}G_{t}^{b}(x,y) \bigr\vert \\& \quad \leq \biggl\vert t\frac{\partial}{\partial x_{j}}G_{t}^{b} \bigl(x',y\bigr)-t\frac{\partial }{\partial x_{j}}G_{t}^{b}(x,y) \biggr\vert + \bigl\vert tx'_{j}G_{t}^{b} \bigl(x',y\bigr)-tx_{j}G_{t}^{b}(x,y) \bigr\vert \\& \quad \doteq J_{1}+J_{2}. \end{aligned}$$

For $J_{2}$, let

$$ \varphi(z)=\varphi_{y,s}(z)=z_{j}\exp{ \biggl(- \frac{1}{4}\alpha (s,z,y) \biggr)}, $$

where $\alpha(s,z,y)=s|z+y|^{2}+\frac{1}{s}|z-y|^{2}$.

Then

$$\frac{\partial\varphi}{\partial z_{k}}(z)= \biggl(\delta_{jk}-\frac {s}{2}z_{j}(z_{k}+y_{k})- \frac{1}{2s}z_{j}(z_{k}-y_{k}) \biggr)\exp{ \biggl(-\frac {1}{4}\alpha(s,z,y) \biggr)}. $$

Therefore

20 $$\begin{aligned} \biggl\vert \frac{\partial\varphi}{\partial z_{k}}(z) \biggr\vert \leq &C\biggl(1+s|z||z+y|+ \frac{1}{s}|z||z-y|\biggr)\exp{ \biggl(-\frac{1}{4}\alpha (s,z,y) \biggr)} \\ \leq&C\biggl(1+s^{1/2}|z|+\frac{1}{s^{1/2}}|z|\biggr)\exp{ \biggl(- \frac{1}{8}\alpha (s,z,y) \biggr)} \\ \leq&C\biggl(1+s^{1/2}\bigl( \vert z-y \vert + \vert z+y \vert \bigr)+\frac{1}{s^{1/2}}\bigl( \vert z-y \vert + \vert z+y \vert \bigr) \biggr) \exp{ \biggl(-\frac{1}{8}\alpha(s,z,y) \biggr)} \\ \leq&C\biggl(1+s+\frac{1}{s}\biggr)\exp{ \biggl(-\frac{1}{16s}|z-y|^{2} \biggr)} \\ \leq&Cs^{-1}\exp{ \biggl(-\frac{1}{16s}|z-y|^{2} \biggr)}. \end{aligned}$$

Let $\theta=\lambda x+(1-\lambda)x'$, $0<\lambda<1$. Then

$$\begin{aligned} J_{2} =&te^{-bt} \bigl\vert x'_{j}K_{s} \bigl(x',y\bigr)-x_{j}K_{s}(x,y) \bigr\vert \\ \leq&Ct^{-d/2} \bigl\vert x-x' \bigr\vert \sup _{\theta} \bigl\vert \nabla\varphi(\theta) \bigr\vert \\ \leq&Ct^{-d/2}\frac{ \vert x-x' \vert }{s}\sup_{\theta}\exp{ \biggl(-\frac{ \vert \theta -y \vert ^{2}}{16s} \biggr)} \\ \leq&Ct^{-d/2}\frac{ \vert x-x' \vert }{t}\sup_{\theta}\exp{ \biggl(-\frac{ \vert \theta -y \vert ^{2}}{16t} \biggr)}. \end{aligned}$$

When $|x-x'|\leq\frac{|x-y|}{2}$, we can get $|\theta-y|\sim|x-y|$. Therefore, there exists $A>0$ such that

21 $$ J_{2}\leq Ct^{-d/2}\frac{|x-x'|}{t}\exp{ \biggl(-\frac{|x-y|^{2}}{At} \biggr)}. $$

For $J_{1}$,

$$\begin{aligned} J_{1} =& \biggl\vert t\frac{\partial}{\partial x_{j}}G_{t}^{b} \bigl(x',y\bigr)-t\frac{\partial }{\partial x_{j}}G_{t}^{b}(x,y) \biggr\vert \\ =&te^{-bt} \biggl\vert \frac{\partial}{\partial x_{j}}K_{s} \bigl(x',y\bigr)-\frac{\partial }{\partial x_{j}}K_{s}(x,y) \biggr\vert \\ =&te^{-bt} \biggl\vert \biggl(s(x_{j}+y_{j})+ \frac{1}{s}(x_{j}-y_{j})\biggr)\exp{ \biggl(- \frac {1}{4}\alpha(s,x,y) \biggr)} \\ &{}-\biggl(s\bigl(x'_{j}+y_{j}\bigr)+ \frac{1}{s}\bigl(x'_{j}-y_{j}\bigr) \biggr)\exp{ \biggl(-\frac{1}{4}\alpha \bigl(s,x',y\bigr) \biggr)} \biggr\vert . \end{aligned}$$

Let

$$\psi(z)=\psi_{y,s}(z)=\biggl(s(z_{j}+y_{j})+ \frac{1}{s}(z_{j}-y_{j})\biggr)\exp{ \biggl(- \frac {1}{4}\alpha(s,z,y) \biggr)}. $$

Then

$$\begin{aligned} \frac{\partial\psi}{\partial z_{k}}(z) =&\biggl[\biggl(s+\frac{1}{s}\biggr) \delta_{jk}-\frac {1}{2}\biggl(s(z_{j}+y_{j})+ \frac{1}{s}(z_{j}-y_{j})\biggr) \\ &{}\times\biggl(s(z_{k}+y_{k})+\frac{1}{s}(z_{k}-y_{k}) \biggr)\biggr]\exp{ \biggl(-\frac{1}{4}\alpha (s,z,y) \biggr)}. \end{aligned}$$

Therefore, similar to the proofs of (20) and (21), we can prove

$$\biggl\vert \frac{\partial\psi}{\partial z_{k}}(z) \biggr\vert \leq Cs^{-1} \exp{ \biggl(-\frac{1}{4}\alpha(s,z,y) \biggr)} $$

and

22 $$\begin{aligned} J_{1} \leq&Ce^{-bt}\sup_{\theta}\bigl\vert \nabla\psi(\theta) \bigr\vert \bigl\vert x-x' \bigr\vert \\ \leq&Ct^{-d/2}\frac{|x-x'|}{t}\exp{ \biggl(-\frac{|x-y|^{2}}{At} \biggr)}. \end{aligned}$$

Inequalities (21) and (22) show

$$ \bigl\vert tA_{j}G_{t}^{b}(x,y)-tA_{j}G_{t}^{b} \bigl(x',y\bigr) \bigr\vert \leq C_{N} \frac{|x-x'|}{t}t^{-\frac{d}{2}}\exp{ \biggl(-\frac {|x-y|^{2}}{At} \biggr)}. $$

Then, similar to the proof of (a), we have

$$\bigl\vert tA_{j}G_{t}^{b}(x,y)-tA_{j}G_{t}^{b} \bigl(x',y\bigr) \bigr\vert \leq C_{N} \frac{|x-x'|}{t}t^{-\frac{d}{2}}\exp{ \biggl(-\frac {|x-y|^{2}}{At} \biggr)} \biggl(1+\frac{\sqrt{t}}{\rho(x)}+\frac{\sqrt {t}}{\rho(y)} \biggr)^{-N}. $$

This completes the proof of Proposition 4. □

The subordination formula gives the following lemma.

### Lemma 3

- *For* $N\in\mathbb {N}$, *there is* $C_{N}>0$ *satisfying*
  23 $$ \bigl\vert tA_{j}P_{t}^{b}(x,y) \bigr\vert \leq C_{N}\frac{t}{(t^{2}+A|x-y|^{2})^{(d+1)/2}} \biggl(1+\frac{t}{\rho(x)}+ \frac{t}{\rho(y)} \biggr)^{-N}. $$
- *For any* $N>0$ *and* $|x-x'|\leq\frac{|x-y|}{2}$, *there are* $C>0$, $C_{N}>0$, *so that*
  24 $$\begin{aligned}& \bigl\vert tA_{j}P_{t}^{b}(x,y)-tA_{j}P_{t}^{b} \bigl(x',y\bigr) \bigr\vert \\& \quad \leq C_{N} \biggl( \frac{|x-x'|}{t} \biggr)\frac{t}{(t^{2}+A|x-y|^{2})^{(d+1)/2}} \biggl(1+\frac{t}{\rho(x)}+ \frac{t}{\rho(y)} \biggr)^{-N}. \end{aligned}$$

## Square function characterizations of $H_{L}^{1}(\mathbb{R}^{d})$

We define square functions

$$\mathcal{G}_{L}^{b,k}f(x)= \biggl( \int_{0}^{\infty} \bigl\vert D_{t}^{b,k}f(x) \bigr\vert ^{2}\frac{dt}{t} \biggr)^{1/2} $$

and

$$S_{L}^{b,k}f(x)= \biggl( \int_{0}^{\infty} \int_{|x-y|< t} \bigl\vert D_{t}^{b,k}f(y) \bigr\vert ^{2}\frac{dy\,dt}{t^{d+1}} \biggr)^{1/2}, $$

where $D_{t}^{b,k}f(x)=t^{k} (\partial_{t}^{k}P_{t}^{b}f )(x)$ for $k=1,2,\ldots $ .

The proof of the following lemma can be found in [4].

### Lemma 4

*If* $f\in L^{1}(\mathbb{R}^{d})$, *we have* $f\in H_{L}^{1}(\mathbb {R}^{d})$ *is equivalent to* $f\in H_{L+b}^{1}(\mathbb {R}^{d})$ *for* $b>0$.

Then, by Lemma 4, we can prove (cf. Section 8 in [11] or [12]) the following.

### Proposition 5

$f\in H_{L}^{1}(\mathbb {R}^{d})$ *is equivalent to its area integral* $S_{L}^{b,k}f\in L^{1}(\mathbb {R}^{d})$ *and* $f\in L^{1}(\mathbb{R}^{d})$. *Moreover*,

$$\|f\|_{H_{L+b}^{1}}\sim\|f\|_{H_{L}^{1}}\sim \bigl\Vert S_{L}^{b,k}f \bigr\Vert _{L^{1}}+\| f \|_{L^{1}}. $$

Motivated by [13], we can prove the following.

### Lemma 5

*There is* $C>0$ *satisfying*

$$\bigl\Vert S_{L}^{b,k+1}f \bigr\Vert _{L^{1}}\leq C \bigl\Vert \mathcal{G}_{L}^{b,k}f \bigr\Vert _{L^{1}}. $$

### Proof

Let

$$F(x) (t)= \bigl(\partial_{t}^{k}e^{-t\sqrt{L+b}}f \bigr) (x),\qquad V(x,s)=e^{-s\sqrt {L+b}}F(x). $$

Then

$$V(x,s) (t)=e^{-s\sqrt{L+b}} \bigl(\partial_{t}^{k}e^{-t\sqrt{L+b}}f \bigr) (x)= \bigl(\partial_{t}^{k}e^{-(s+t)\sqrt{L+b}}f \bigr) (x). $$

Therefore

$$\begin{aligned} \int_{0}^{+\infty} \bigl\vert V(x,s) (t) \bigr\vert ^{2}t^{2k-1} \,dt =& \int_{0}^{+\infty} \bigl\vert \bigl( \partial_{t}^{k}e^{-(s+t)\sqrt{L+b}}f \bigr) (x) \bigr\vert ^{2}t^{2k-1}\,dt \\ =& \int_{s}^{+\infty} \bigl\vert \bigl( \partial_{t}^{k}e^{-t\sqrt{L+b}}f \bigr) (x) \bigr\vert ^{2}(t-s)^{2k-1} \,dt. \end{aligned}$$

Hence

$$\sup_{s>0} \int_{0}^{+\infty} \bigl\vert V(x,s) (t) \bigr\vert ^{2}t^{2k-1}\,dt \leq \int_{0}^{+\infty} \bigl\vert \bigl(t^{k} \partial_{t}^{k}e^{-t\sqrt{L+b}}f \bigr) (x) \bigr\vert ^{2}\frac{dt}{t} =\bigl(\mathcal{G}_{L}^{b,k}f(x) \bigr)^{2}. $$

Let $\mathbf{X}=L^{2} ((0,\infty), t^{2k-1}\,dt )$. Then

$$\sup_{s>0} \bigl\Vert e^{-s\sqrt{L+b}}F(x) \bigr\Vert _{\mathbf{X}}=\mathcal {G}_{L}^{b,k}f(x)\in L^{1}\bigl(\mathbb {R}^{d}\bigr). $$

Therefore $F\in H_{\mathbf{X}}^{1}(\mathbb {R}^{d})$, here $H_{\mathbf{X}}^{1}(\mathbb {R}^{d})$ is a vector-valued Hardy space. Therefore $\widetilde{S_{L}^{b,1}}F(x)\in L^{1}(\mathbb {R}^{d})$, where

$$\widetilde{S_{L}^{b,1}}F(x)= \biggl( \int_{0}^{+\infty} \int_{|z-y|< 2t} \bigl\Vert D_{t}^{b,1}F(y) \bigr\Vert _{\mathbf{X}}^{2}\frac{dy\, dt}{t^{d+1}} \biggr)^{1/2}. $$

By

$$\begin{aligned} \bigl(\widetilde{S_{L}^{b,1}}F(x)\bigr)^{2} =& \int_{0}^{+\infty} \int_{|x-y|< 2t} \bigl\Vert D_{t}^{b,1}(x) \bigr\Vert _{\mathbf {X}}^{2}\frac{dy \,dt}{t^{d+1}} \\ =& \int_{0}^{+\infty} \int_{ \vert x-y \vert < 2t} \int_{0}^{+\infty} \bigl\vert (-t\sqrt{L+b})e^{-t\sqrt{L+b}}F(y) (s) \bigr\vert ^{2}s^{2k-1}\,ds\frac{dy \,dt}{t^{d+1}} \\ =& \int_{0}^{+\infty} \int_{0}^{+\infty} \int_{ \vert x-y \vert < 2t} \bigl\vert (-\sqrt{L+b})^{k+1}e^{-(s+t)\sqrt{L+b}}f(y) \bigr\vert ^{2} \\ &{}\times t^{1-d}s^{2k-1}\,dy\,dt\,ds \\ =& \int_{0}^{+\infty} \int_{s}^{+\infty} \int_{ \vert x-y \vert < 2(t-s)} \bigl\vert (-\sqrt{L+b})^{k+1}e^{-t\sqrt{L+b}}f(y) \bigr\vert ^{2} \\ &{}\times (t-s)^{1-d}s^{2k-1}\,dy\,dt\,ds \\ =& \int_{0}^{+\infty} \int_{0}^{t} \int_{ \vert x-y \vert < 2(t-s)} \bigl\vert (-\sqrt{L+b})^{k+1}e^{-t\sqrt{L+b}}f(y) \bigr\vert ^{2} \\ &{}\times (t-s)^{1-d}s^{2k-1}\,dy\,ds\,dt \\ \geq& \int_{0}^{+\infty} \int_{0}^{t/2} \int_{ \vert x-y \vert < 2(t-s)} \bigl\vert (-\sqrt{L+b})^{k+1}e^{-t\sqrt{L+b}}f(y) \bigr\vert ^{2} \\ &{}\times (t-s)^{1-d}s^{2k-1}\,dy\,ds\,dt \\ \geq& \int_{0}^{+\infty} \int_{0}^{t/2} \int_{ \vert x-y \vert < t} \bigl\vert (-\sqrt{L+b})^{k+1}e^{-t\sqrt{L+b}}f(y) \bigr\vert ^{2}t^{1-d}s^{2k-1}\,dy\,ds\,dt \\ =&\frac{1}{2k2^{2k}} \int_{0}^{+\infty} \int_{ \vert x-y \vert < t} \bigl\vert (-t\sqrt {L+b})^{k+1}e^{-t\sqrt{L+b}}f(y) \bigr\vert ^{2} t^{-1-2n}\, dy\, dt \\ =&\frac{1}{2k2^{2k}} \int_{0}^{+\infty} \int_{ \vert x-y \vert < t} \bigl\vert D_{t}^{b,k+1}f(y) \bigr\vert ^{2}\frac{dy \,dt}{t^{d+1}}=\frac{1}{2k2^{2k}} \bigl(S_{L}^{b,k+1}f(x)\bigr)^{2}, \end{aligned}$$

we get $\|S_{L}^{b,k+1}f\|_{L^{1}}\leq C \|\mathcal{G}_{L}^{b,k}(f)\|_{L^{1}}$. □

By Lemma 5, we can prove the following.

### Proposition 6

$f\in H_{L}^{1}(\mathbb {R}^{d})$ *is equivalent to* $\mathcal {G}_{L}^{b,k}f\in L^{1}(\mathbb {R}^{d})$ *and* $f\in L^{1}(\mathbb{R}^{d})$. *Moreover*,

$$\|f\|_{H_{L+b}^{1}}\sim \bigl\Vert \mathcal{G}_{L}^{b,k}f \bigr\Vert _{L^{1}}+\|f\|_{L^{1}}. $$

Similar to the proof of Lemma 14 in [9], we have the following.

### Lemma 6

*Let* *a* *be an* $H_{L}^{1,\infty}$-*atom*. *Then we can find a constant* $C>0$ *satisfying*

$$\bigl\Vert \mathcal{G}_{b}(a) \bigr\Vert _{L^{1}}\leq C. $$

As pointed out in [14], we cannot get that an operator is bounded on $H_{L}^{p}(\mathbb {R}^{d})$ by just proving that it is uniformly bounded on atoms. But we have the following lemma (cf. p.316, Theorem 7.3 in [15]).

### Lemma 7

*Let* *T* *be an integral operator with the kernel in the Campanato space* $\Lambda_{d(1/p-1)}$ *and satisfy* $\|Ta\|_{L^{p}}\leq C $ *for all the* $H_{L}^{p,q}$-*atom* $a(x)$, *then* *T* *is a bounded operator from* $H_{L}^{p}(\mathbb {R}^{d})$ *to* $L^{p}(\mathbb {R}^{d})$.

In the following, we prove $D_{t}^{b}(x,y)=tA_{j}P_{t}^{b}(x,y)$ belongs to $\mathit{BMO}_{L}$, which is defined in [8].

### Lemma 8

*For every* $t>0$ *and* $x\in\mathbb{R}^{d}$, *we have* $D_{t}^{b}(x,y)\in \mathit{BMO}_{L}$.

### Proof

For any ball $B(y_{0},r)$, if $r<\rho(y_{0})$ and $r< t$, then by Lemma 3(b) we have

25 $$\begin{aligned}& \frac{1}{|B|^{1/2}} \biggl( \int_{B} \bigl\vert D_{t}^{b}(x,y)-D_{t}^{b}(x,y_{0}) \bigr\vert ^{2}\,dy \biggr)^{1/2} \\& \quad \leq C r^{-d/2} \biggl( \int_{B} \biggl(\frac{|y-y_{0}|}{t} \biggr)^{2} \frac{t^{-2d}}{(1+t^{-2}|x-y_{0}|^{2})^{d+1}}\,dy \biggr)^{1/2} \\& \quad \leq C t^{-d}\biggl(\frac{r}{t}\biggr)\leq C t^{-d}. \end{aligned}$$

If $t\leq r<\rho(y_{0})$, then by Lemma 3(a)

26 $$ \frac{1}{|B|^{1/2}} \biggl( \int_{B} \bigl\vert D_{t}^{b}(x,y)-D_{t}^{b}(x,y_{0}) \bigr\vert ^{2}\,dy \biggr)^{1/2}\leq C t^{-d}. $$

If $r\geq\rho(y_{0})$, then by Lemma 3(a) we have

27 $$\begin{aligned} \begin{aligned}[b] &\frac{1}{|B|^{1/2}} \biggl( \int_{B} \bigl\vert D_{t}^{b}(x,y) \bigr\vert ^{2}\,dy \biggr)^{1/2} \\ &\quad \leq C r^{-d/2} \biggl( \int_{B} \frac{t^{-2d}}{(1+t^{-2}|x-y_{0}|^{2})^{d+1}}\,dy \biggr)^{1/2} \\ &\quad \leq C t^{-d}r^{-d/2}|B|^{1/2}\leq Ct^{-d}. \end{aligned} \end{aligned}$$

Then Lemma 8 follows from (25)-(27). □

Now, let us prove Theorem 1.

### Proof of Theorem 1

When $f\in L^{1}(\mathbb{R}^{d})$ and $\mathcal{G}_{b}(f)\in L^{1}(\mathbb {R}^{d})$, by Proposition 5 and Lemma 5, we have

$$\begin{aligned} \Vert f \Vert _{H_{L}^{1}} \leq&C \Vert f \Vert _{H_{L+b}^{1}}\leq C \bigl\{ \bigl\Vert S_{L}^{b,2}(f) \bigr\Vert _{L^{1}}+ \Vert f \Vert _{L^{1}}\bigr\} \leq C\bigl\{ \bigl\Vert \mathcal{G}_{L}^{b,1}(f) \bigr\Vert _{L^{1}}+ \Vert f \Vert _{L^{1}}\bigr\} \\ \leq&C\bigl\{ \bigl\Vert \mathcal{G}_{b}(f) \bigr\Vert _{L^{1}}+ \Vert f \Vert _{L^{1}}\bigr\} . \end{aligned}$$

Therefore, $f\in H_{L}^{1}(\mathbb {R}^{d})$.

The reverse can be proved by Lemmas 6, 7 and 8.

Theorem 1 is proved. □

## Riesz transform associated with *L*

We introduce the following version of Riesz transform:

$$R_{j}^{L,b}=A_{j}(L+b)^{-1/2},\quad j=1,2,\ldots,d, b>0. $$

If $f\in L^{2}(\mathbb{R}^{d})$, then

28 $$\begin{aligned} R_{j}^{L,b}f =&\sum_{\alpha}\biggl(\frac{2\alpha_{j}}{2|\alpha|+d+b} \biggr)^{1/2} \langle f, h_{\alpha}\rangle h_{\alpha-e_{j}} \\ =&\sum_{n=0}^{\infty}\sum _{|\alpha|=n} \biggl(\frac{2\alpha _{j}}{2n+d+b} \biggr)^{1/2} \langle f, h_{\alpha}\rangle h_{\alpha-e_{j}}. \end{aligned}$$

We can prove the following.

### Theorem 3

*Let* $j=1,2,\ldots,d$. *Then* $R_{j}^{L,b}$ *are bounded operators on* $H_{L}^{1}(\mathbb {R}^{d})$, *that is*, *there is* $C>0$ *satisfying*

$$\bigl\Vert R_{j}^{L,b}f \bigr\Vert _{H_{L}^{1}}\leq C \|f \|_{H_{L}^{1}}. $$

### Proof

When $f\in L^{2}(\mathbb{R}^{d})$, following (28), it is not difficult to check

29 $$ D_{t}^{b+2,1}R_{j}^{L,b}f=-tA_{j}u_{b}(x,t) $$

for $j=1,2,\ldots,d$. As $H_{L}^{1}(\mathbb{R}^{d})\cap L^{2}(\mathbb{R}^{d})$ is dense in $H_{L}^{1}(\mathbb{R}^{d})$ (see [11]), we can assume $f\in H_{L}^{1}(\mathbb{R}^{d})\cap L^{2}(\mathbb{R}^{d})$. Then, by Lemma 5, Theorem 1, Proposition 6 and (29), we get

$$\begin{aligned} \bigl\Vert R_{j}^{L,b}f \bigr\Vert _{H_{L}^{1}} \leq&C \bigl\Vert R_{j}^{L,b}f \bigr\Vert _{H_{L+b+2}^{1}} \leq C \bigl\Vert \mathcal{G}_{L}^{b+2,1}\bigl(R_{j}^{L,b}f \bigr) \bigr\Vert _{L^{1}} \\ =&C \biggl\Vert \biggl( \int_{0}^{\infty}\bigl|tA_{j}u_{b}(x,t)\bigr|^{2} \frac{dt}{t} \biggr)^{1/2} \biggr\Vert _{L^{1}} \\ \leq&C \bigl\Vert \mathcal{G}_{b}(f) \bigr\Vert _{L^{1}} \leq C \Vert f \Vert _{H_{L}^{1}}. \end{aligned}$$

This proves Theorem 3. □

The proof of the following lemma can be found in [16].

### Lemma 9

*If* $\beta\in\mathbb {R}$ *and* $f\in L^{2}(\mathbb {R}^{d})$, *then*

$$A_{j}L^{\beta}f=(L+2)^{\beta}A_{j}f, $$

*for* $j=1,2,\ldots,d$.

Now, we can prove Theorem 2.

### Proof of Theorem 2

Since $H_{L}^{1}(\mathbb{R}^{d})\cap L^{2}(\mathbb{R}^{d})$ is dense in $H_{L}^{1}(\mathbb{R}^{d})$ (see [11]), we can assume $f\in H_{L}^{1}(\mathbb{R}^{d})\cap L^{2}(\mathbb{R}^{d})$. We prove Theorem 2 by an inductive argument.

When $m=1$, Theorem 2 has been proved in [4]. We assume that Theorem 2 holds for $m-1$, by Lemma 9 and Theorem 3,

$$\begin{aligned} \bigl\Vert R_{i_{1}i_{2}\cdots i_{m}}L^{-m/2}f \bigr\Vert _{H_{L}^{1}} =& \bigl\Vert A_{i_{1}}\bigl(L+2(m-1)\bigr)^{-1/2}A_{i_{2}} \cdots A_{i_{m}}L^{-(m-1)/2}f \bigr\Vert _{H_{L}^{1}} \\ \leq& \bigl\Vert A_{i_{2}}\cdots A_{i_{m}}L^{-(m-1)/2}f \bigr\Vert _{H_{L}^{1}}\leq \Vert f \Vert _{H_{L}^{1}}. \end{aligned}$$

Therefore Theorem 2 holds. □

## Conclusions

In this paper, we consider the Riesz transforms of higher order associated with a harmonic oscillator and prove the boundedness of them on the Hardy space. It is well known that the Riesz transforms play an important role in the study of harmonic analysis and partial differential equations. These results are very good progress on the harmonic analysis of Hermite operators.
